# Kinesio Taping effects on knee extension force among soccer players

**DOI:** 10.1590/bjpt-rbf.2014.0075

**Published:** 2015-03-13

**Authors:** Maysa V. G. B. Serra, Edgar R. Vieira, Denis Brunt, Márcio F. Goethel, Mauro Gonçalves, Paulo R. V. Quemelo

**Affiliations:** 1 Departamento de Promoção da Saúde, Universidade de Franca (UNIFRAN), Franca, SP, Brazil; 2 Departamento de Fisioterapia, UNIFRAN, Franca, SP, Brazil; 3 Department of Physical Therapy, Florida International University, Miami, FL, United States; 4 Departamento de Educação Física, Instituto de Biociências, Universidade Estadual de São Paulo (UNESP), Rio Claro, SP, Brazil

**Keywords:** Kinesio Taping, athletes, athletic performance, muscle strength, physical therapy

## Abstract

**Background:**

Kinesio Taping (KT) is widely used, however the effects of KT on muscle activation and force are contradictory.

**Objective:**

To evaluate the effects of KT on knee extension force in soccer players.

**Method:**

This is a clinical trial study design. Thirty-four subjects performed two maximal isometric voluntary contractions of the lower limbs pre, immediately post, and 24 hours after tape application on the lower limbs. Both lower limbs were taped, using K-Tape and 3M Micropore tape randomly on the right and left thighs of the participants. Isometric knee extension force was measured for dominant side using a strain gauge. The following variables were assessed: peak force, time to peak force, rate of force development until peak force, time to peak rate of force development, and 200 ms pulse.

**Results:**

There were no statistically significant differences in the variables assessed between KT and Micropore conditions (*F*=0.645, *p*=0.666) or among testing sessions (pre, post, and 24h after) (*F*=0.528, *p*=0.868), and there was no statistical significance (*F*=0.271, *p*=0.986) for interaction between tape conditions and testing session.

**Conclusion:**

KT did not affect the force-related measures assessed immediately and 24 hours after the KT application compared with Micropore application, during maximal isometric voluntary knee extension.

## Introduction

Soccer is the most popular sport in the world. It is played in almost every country, with an estimated 265 million participants^1^. Soccer players require a large range of motor skills, as well as the need for rapid information processing and decision-making as play moves between attacking and defending actions. Resistance exercise and different kinds of training (balance and coordination) are responsible for improving velocity and movement coordination, in addition to promoting the balance and functionality needed to achieve significant and visible improvements in strength and muscular performance^2^.

Kinesio Taping (KT) is often used by soccer players to improve performance during training and competitions^3^. KT is a high-viscosity, adhesive elastic tape that allows the skin to breathe and is water resistant. According to Kase et al.^4^, KT provides constant mechanical/elastic stimulation of the skin, and its effects are transmitted to deeper tissues through mechanoreceptors found in the epidermis and dermis. Others suggest that KT may modify the muscle activity and increase force^5^^-^ 7. Słupik et al.^8^ evaluated 27 healthy subjects with mean age of 23 (standard deviation [SD] 3.5) years and found an increase in muscle activity of the vastus medialis 24 hours after KT application, and the effect remained for 48 hours after application. Huang et al.^9^ found an increase in triceps surae muscle activity during vertical jump post KT application in 31 healthy adults (19 males and 12 females) with mean age of 25 (SD 4) years. However, other studies found that applying KT did not alter knee extension force when applied over the quadriceps muscle in healthy athletes^10^^-^ 12. Also, KT did not improve jump performance or balance in healthy college athletes^13^. In addition, three recent systematic reviews identified few high-quality studies and those studies provided insufficient evidence to support the use of KT in clinical conditions^14^^-^ 16.

Despite being commonly used, the results of the studies that evaluated the effects of KT on muscle activation and force in athletes are contradictory. It has been proposed that KT application may cause a small immediate increase in muscle strength by pulling on the fascia, stimulating increased muscle contraction^3^^,^ 4. However, empirical evidence is lacking and further evaluation of the effects of KT application on force exertion is needed^17^. Therefore, the objective of this study was to evaluate the effect of KT on knee extension force in soccer players. The study hypothesis was that KT application would improve knee extension force in soccer players.

## Method

### Subjects

**Figure 1.**
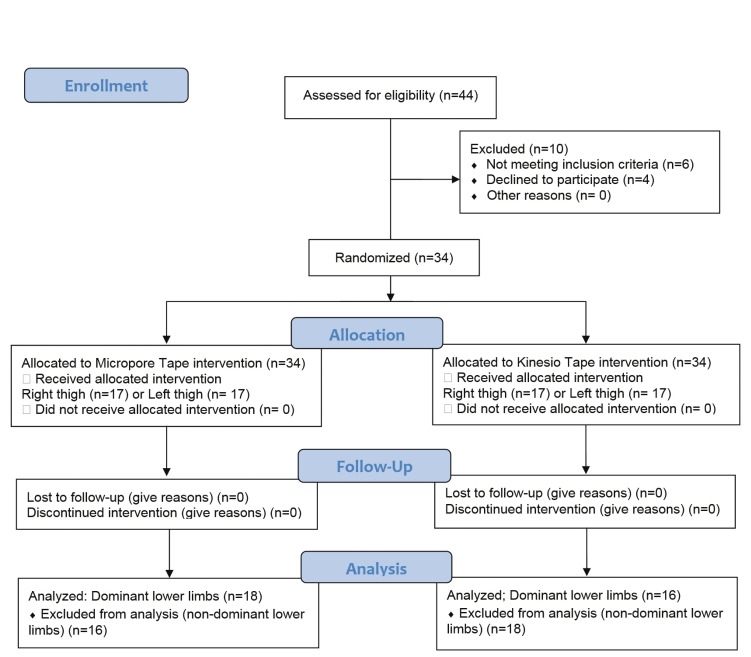
Flow diagram of soccer players during of the study.

This was a clinical trial. Figure 1 presents the study flow diagram. The participants were recruited from the male and female professional soccer teams of the city of Franca, SP, Brazil. Forty-four players were invited to participate in study. The inclusion criteria were to be a soccer player for more than one year and be at least 18 years old. The exclusion criterion was having lower limb injuries at the time of testing. Thirty-four (77%) professional soccer players (20 males and 14 females) were eligible and volunteered to participate in the study. The sample size was calculated using GPower 3.0 software and at least 30 subjects would be needed to have a power of 0.82 to identify differences between the two groups. The study was approved by the institutional review board from the Universidade de Franca (UNIFRAN), Franca, SP, Brazil (protocol # 009/11) and all participants signed an informed consent form.

### Equipment

The isometric knee extension force was collected using a strain gauge (EMG System do Brasil Ltda.^(r))^ with a measuring range from 0 to 200 kg at 1000 Hz. The data was filtered with a fourth-order Butterworth low-pass filter and cut-off frequency of 15 Hz, obtained through residual analysis. The equipment (strain gauge) was attached to the extensor chair lever arm and the chair's legs.

### Procedures and data collected

Force testing: The study was conducted in a university laboratory setting. The data was collected before the athletes' morning training. The participants were seated on a knee extensor chair, with the back straight, arms crossed, and knees bent at 90º^18^. Velcro straps/belts were used to stabilize the participants. The lever arm of the extensor chair was positioned near the ankle above the malleoli. The testing position followed the procedures proposed for rectus femoris muscle function test by Kendall et al.^19^. The participants performed two five-second maximal isometric voluntary knee extension trials^20^ using each of the lower limbs during three testing sessions: pre, immediately post, and 24 hours after application of the tape (KT and Micropore conditions). A rest interval of 2 minutes was given to the participants between trials to prevent fatigue^21^. After the testing session, the participants were instructed to continue their normal activities, but were asked not to wash or remove the tapes. Twenty-four hours later, the participants returned to the lab for one additional testing session following the same procedures. All 34 soccer players completed all tests with both lower limbs (KT and Micropore conditions) during all testing sessions (pre, post and 24h after tape application).

**Figure 2.**
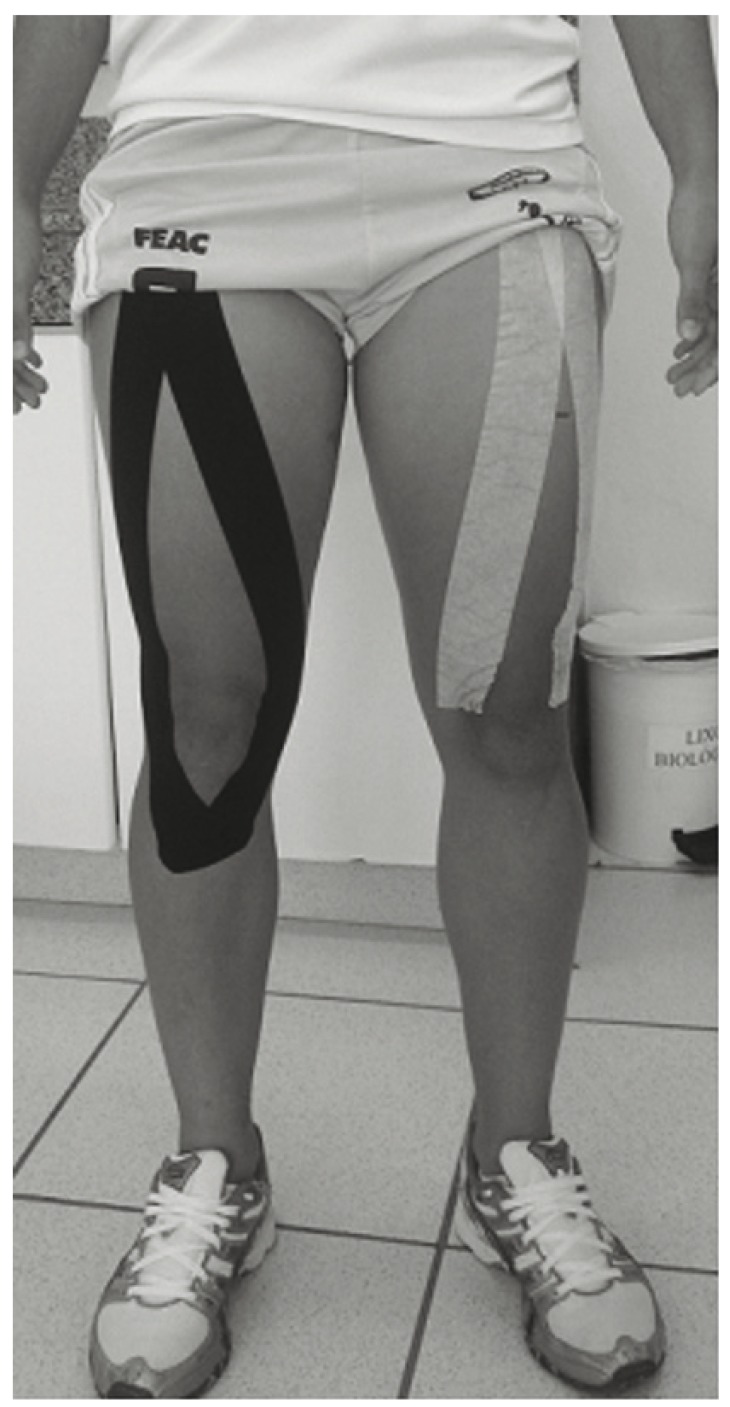
Application of Kinesio Tape (right lower limb) and 3M Micropore ® (left lower limb).

Tape application: After the pre-testing session (no tape condition), KT (K-Tape^(r)^ brand) was applied on the skin over the rectus femoris muscle on one limb and 3M Micropore^(r)^ (placebo tape) was applied on the contralateral limb. First, the sites of tape placement were shaved and cleaned with 70% rubbing alcohol. The tapes were randomly placed on the right and left lower limbs over the quadriceps muscle. Therefore, both limbs were taped, one side with Micropore and the other with KT in random order among participants. Half of the participants (n=17) had KT applied to the left thigh and the other half to the right thigh (n=17). KT was applied using the "V" technique, the knee was positioned at 45º of flexion, the origin of both tapes was located 10 cm below the anterior-superior iliac spine with one tape going laterally and one medially to the rectus femoris muscle belly, passing around the patella and finishing on the tibial tuberosity^22^^,^ 23. The same technique was used for the Micropore tape on the contralateral limb, but because it is not elastic, the knee joint was not crossed and the end points were the medial and lateral aspects of the patella (Figure 2). After the final testing session the tapes were removed and the skin was cleaned with rubbing alcohol.

### Data analysis

After data collection, the results for each subject's dominant side were divided into KT group (n=16; dominant lower limb) and Micropore tape group (n=18 dominant lower limb). The mean force values were calculated just for the dominant side for each condition (pre, post and 24 hours after tape application) and each tape (KT and Micropore).

We assessed the following force-related variables:

- Peak force: maximum amount of force during the trials normalized by each participant's body mass;
- Time to peak force: time in seconds elapsed between onset of force and peak of force;
- Rate of force development until peak force: rate of force increase until peak force was reached;
- Time to peak rate of force development: time in seconds when the peak rate of force development was observed;
- 200 ms pulse: Force/time plot area for the initial 200 ms after force onset.

### Statistical analysis

Descriptive statistics (mean and SD) were calculated. The normalized data for males and females were combined because there were no differences between the genders after normalization. The variables were compared between KT and Micropore conditions, and among testing sessions (pre, post, and 24h after), and the interaction effects between tape conditions and testing sessions (time) were assessed using repeated measure MANOVA. All analyses were conducted using *Statistica* (version 8.0 - StatSoft, Inc. 2007), with the level of significance set at 0.05.

## Results

The participants' demographics are presented in Table 1by gender. The force-related variables measured during maximum isometric knee extension are presented in Table 2. The 200 ms pulse for 24 hours after KT application (M=87; [SD=25.3]) tended to be higher with KT than with Micropore tape (M=76.1; [SD=21.1]). However, there were no statistically significant differences between the KT and Micropore conditions (*F*=0.645, *p*=0.666) or among testing sessions (pre, post, and 24h after) (*F*=0.528, *p*=0.868), and there was no significant interaction between tape condition and testing session (*F*=0.271, *p*=0.986) (Table 3).

**Table 1.** Description of the participants.

Variables	Females (N=14)	Males (N=20)	Total (N=34)
Age (years old)	24 (4)	22 (3)	23 (1)
Height (cm)	165 (4)	180 (5)	172 (10)
Weight (Kg)	59 (6)	77 (7)	68 (12)
BMI (Kg/m²)	22 (2)	24 (1)	23 (2)
Soccer experience (years)	5 (2)	4 (2)	4 (1)

Mean and (standard deviation).

**Table 2.** Comparison of the force-related variables measured during maximum voluntary isometric knee extension testing sessions completed pre, post, and 24h after hours after kinesio tape (KT) and Micropore tape application.

Force-related variable	Pre-Application		Post-Application		24h After Application	
	Micropore^*^	KT^*^	Micropore	KT	Micropore	KT
Peak force	1.03 (0.19) [0.93-1.12]	1.04 (0.18) [0.94-1.13]	1.02 (0.18) [0.93-1.11]	1.03 (0.17) [0.94-1.11]	0.95 (0.21) [0.85-1.05]	1.01 (0.15) [0.92-1.09]
Time to peak force	3.39 (0.96) [2.91-3.86]	2.97 (1.08) [2.40-3.55]	3.35 (0.94) [2.88-3.81]	3.21 (1.02) [2.66-3.75]	3.19 (1.08) [2.65-3.73]	3.32 (0.99) [2.78-3.84]
Time to peak rate of force development	0.10 (0.05) [0.08-0.12]	0.08 (0.03) [0.06-0.10]	0.11 (0.06) [0.07-0.13]	0.10 (0.05) [0.07-0.13]	0.12 (0.06) [0.08-0.14]	0.10 (0.04) [0.07-0.012]
Rate of force development until peak	2.4 (1.6) [1.59-3.22]	3 (2.2) [1.79-4.20]	2.4 (2.1) [1.35-3.51]	2.6 (1.5) [1.85-3.46]	2.2 (2.2) [1.10-3.28]	2.67 (1.5) [1.88-3.46]
200 ms pulse	76.6 (22.3) [65.53-87.72]	84.5 (34) [66.36-102.6]	83.7 (26.3) [70.61-96.75]	82.1 (21.3) [70.71-93.43]	76.1 (21.1) [65.65-86.65]	87.1 (25.3) [73.63-100.6]

* The pre-application values are from the legs without tape that subsequently received the respective types of tape. Mean, (standard deviation) and [95% CI - Confidence Interval].

**Table 3.** Multivariate tests of significance, effect sizes, and powers for tape conditions (Micropore vs Kinesio Taping), time sessions (pre, post, and 24h hours) and interaction effect between tape and time using MANOVA-repeated measures.

Variables	Wilkis Test	F	P	Partial Eta-squared	Non-centrality	Power^a^
Types of Tape	0.966140	0.645	0.6660	0.0338	3.22	0.2249
Time sessions	0.944951	0.528	0.8688	0.0279	5.28	0.2695
Interaction effect of the Tape vs Time	0.971135	0.271	0.9866	0.0145	2.71	0.1463

F Statistic; P values; a= alpha= 0.05.

## Discussion

Immediately after KT application, the rate of force development until peak tended to be slightly higher. In the same way, it was observed that 24 hours after KT application the rate of force development until peak and 200 ms pulse tended to be slightly higher than Micropore tape. However, KT did not significantly change knee extension force in healthy athletes immediately post or 24 h after application in relation to pre-application (no tape condition), and there were no differences between the side with KT and the side with Micropore tape, and there was no interaction between kind of tape and time sessions by MANOVA test. Other studies also did not find significant differences immediately after KT application^23^^-^ 25. Chang et al.^26^ found no change in grip strength immediately after applying KT in healthy people, but found better force reproducibility among subjects with KT. Lins et al.^27^ evaluated the effects of KT application on the activity of the vastus lateralis, rectus femoris, and vastus medialis muscles of healthy women who exercised and found no significant effects. Słupik et al.^8^ evaluated the effects of applying KT over the vastus medialis and, similarly to us, found no change in muscle activity 10 minutes post-taping but, unlike us, they found increased muscle activity 24 hours after KT application. The difference between these results may be due to different forms and tensions of KT application. Different KT techniques can provide different tactile stimuli intensities^4^.

In this study we need to considerate some limitations: the participants were not evaluated by a blinded assessor; the isometric knee extension was evaluated only at 90º; the sample was small. On the other hand, we evaluated healthy professional soccer players, which can be considered a good and homogeneous sample.

Huang et al.^9^ evaluated thirty-one healthy adults and found increased stabilization (based on ground reaction forces) and protection (increased muscle activity/EMG), and decreased ankle joint motion (using a video-based motion analysis system) during vertical jumps after KT application. Also differently from our findings, Vithoulka et al.^28^ found increased eccentric knee extension force in healthy adults post KT application over the skin of the quadriceps. The difference between the results may be due to differences in the populations evaluated and testing procedures (e.g. contraction duration and type of contraction), but there may be differences between KT effects during isometric and isotonic/isokinetic testing. Future studies should evaluate this hypothesis.

It must be taken into account that our sample was composed of soccer players without injuries. Most previous studies included injured subjects undergoing rehabilitation^11^^,^ 22 ^,^ 29. Hsu et al.^22^ found significant improvements in scapular motion in baseball players with shoulder impingement after KT application. Thelen et al.^11^ evaluated subjects with rotator cuff tendonitis and found decreased pain immediately after KT application. Thus, KT may have some beneficial effects in patients with motion disorders and pain, even though we found it did not increase knee extension force in healthy subjects.

Kim and Lee^23^ evaluated the effectiveness of KT to prevent injury and improve the performance of 8 horse racing jockeys. The participants performed knee flexion/extension at 60º/sec and 180º/sec in an isokinetic dynamometer before and after KT application, and significant differences were found. Our study used a strain gauge and evaluated only isometric knee extension at 90º of flexion. Thus, it is possible that KT has an effect during motion but not during isometric contractions. Further studies are also needed to evaluate this other hypothesis.

Although it has been theorized that KT increases circulation and subsequently improving muscle function, a recent study with sixty-one healthy people showed that KT application did not affect circulation or volume of the gastrocnemius muscle and does not enhance anaerobic muscle function^30^. Another physiological theory about KT effects is motoneuron activation. A push start on a motoneuron propagates in the axon membrane and affects muscle, giving rise to a flow-reversing ionic resting membrane potential, causing the muscular action potential^31^^,^ 32. Although KT can provide sensory stimuli to the skin and possibly to the underlying fascia through interconnections between the connective tissues, resulting in better motor response and increased muscle contraction^4^^,^ 8 ^,^ 22, the changes that we found were not significant even though the variability was not high, showing that even after 24 hours the parameters remained the same or with non-significant changes. The lack of differences between the sides with KT and Micropore tape indicate that any effect may be due to the skin stimulation independently of the elasticity of the tape or due to placebo effects. Thus, clinically KT should not be used by healthy professional athletes with the aim of improving force.

## Conclusion

KT did not affect the force-related measures assessed immediately and 24 hours after the KT application compared with Micropore application, during maximal isometric voluntary knee extension trials performed by healthy professional athletes.
